# Facile design of lidocaine-loaded polymeric hydrogel to persuade effects of local anesthesia drug delivery system: complete *in vitro* and *in vivo* toxicity analyses

**DOI:** 10.1080/10717544.2021.1931558

**Published:** 2021-06-11

**Authors:** Yan Li, Erxian Zhao, Li Li, Liying Bai, Wei Zhang

**Affiliations:** Department of Anesthesiology, The First Affiliated Hospital of Zhengzhou University, Zhengzhou, China

**Keywords:** Hydrogel, LDC, *in vitro* release, L929 cells, *in vivo* evaluations, drug delivery

## Abstract

The principal goal of the present investigation was to enterprise new and effective drug delivery vesicle for the sustained delivery of local anesthetic lidocaine hydrochloride (LDC), using a novel combination of copolymeric hydrogel with tetrahydroxyborate (COP–THB) to improve bioactivity and therapeutic potential. To support this contention, the physical and mechanical properties, rheological characteristics, and component release of candidate formulations were investigated. An optimized formulation of COP–THB containing LDC to an upper maximum concentration of 1.5% w/w was assessed for drug crystallization. The biocompatibility of the prepared COP–THB hydrogel was exhibited strong cell survival (96%) and growth compatibility on L929 fibroblast cell lines, which was confirmed by using methods of MTT assay and microscopic observations. The COP–THB hydrogel release pattern is distinct from that of COP–THB/LDC hydrogels by the slow-release rate and the low percentage of cumulative release. *In vivo* evaluations were demonstrated the anesthetic effects and toxicity value of treated samples by using mice models. In addition, COP–THB/LDC hydrogels significantly inhibit in vivo tumor growth in mice model and effectively reduced it is *in vivo* toxicity. The pharmacological evaluation showed that encapsulation of LDC in COP–THB hydrogels prolonged its anesthetic action with favorable *in vitro* and *in vivo* compatibility. This novel design may theoretically be used in promising studies involving the controlled release of local anesthetics.

**Highlights**
- Development a modified sustained release system for the local anesthetic lidocaine.
- PVP-THB hydrogel to improve the pharmacological properties of the drug and their anesthetic activities.
- Profiles of PVP-THB/LDC showed that the effective release of associated lidocaine.
- This new formulation could potentially be used in future local anesthetics.

## Introduction

There is a compelling need for prolonged local anesthetic that would be used for analgesia with a single administration (Ma et al., 2017). However, due to the low molecular weight of local anesthetics (lidocaine, bupivacaine, procaine, dibucaine, etc.), they present rapid universal fascination (Becker & Reed, 2006). Local anesthetics are drugs that facilitate the adjustable blocking of neural conduction throughout peripheral nerves by constraining excitation–transmission mechanism. Drugs interrelate with sodium channels in both ionized and non-ionized systems, controlling the membrane probable by blocking nervous conduction, according to recent research (Fozzard et al., 2011). The field of research has mainly integrating drugs inside nanosystems in addition to extending the anesthetic effect, minimizing toxicity, and allowing the processing of high doses of drug (Li et al., 2019). Lidocaine was the active agent and was used as a standard molecule for hydrophobic drug binding and release studies (Weinberg, 2015). Specifically, lidocaine hydrochloride (LDC) is preferred for interesting application since of rapid onset and an extensive duration of action (Abu-Huwaij et al., 2007). LDC is an amide of the local anesthetic form, which, as of its small molecular weight and low water solubility, can be regarded as a perfect molecule for hydrophobic drug binding. Because of its efficacy, rapid controlled release, mild duration of effects, and topical anesthetic activity, it is the most commonly used drug (Becker & Reed, 2012). LDC can cause adverse structural reactions in the nervous and immune system, such as vasodilation, activation of serious arrhythmias, and decreased cardiac contractility, just like any other drug. This drug is a local anesthetic, typically used to alleviate discomfort or itch associated with acute or skin pathologies and LDC was loaded into the hydrogels. In addition, for the diagnosis of aphthae or other painful mouth diseases, LDC-loaded hydrogels may be considered (Bagshaw et al., 2015).

Hydrogels are of particular interest in tissue management because of their low toxicity and potential for extended drug release (Ahmed, 2015). Hydrogels based on copolymer (COP) using polyvinylpyrrolidone (PVP)- and Poly(vinyl alcohol) (PVA)-based hydrogels have several of these beneficial qualities, as well as special frictional flow and mechanical strength, in drug delivery technology (Razzak et al., 2001). COP is a biodegradable, water-soluble polymer with excellent biocompatibility. With a variety of metal ions and charged secondary diazo dyes, it forms ion complexes (Husain et al., 2018). Hydrogels based on COP and one of a variety of cross-linkers, such as tetrahydroxyborate (THB) anions, are one promising class of hydrogels (Al-Emam et al., 2020). The amount of THB anions has a greater effect on hydrogel construction than the amount of COP, according to results from formulation studies. The second step of the complex reaction is aided by sodium ions generated from the detachment of sodium tetraborate, which is a big component of aqueous THB. Sodium ions attenuate the overall negative charge on the polyelectrolyte chain, which aids the second step of the complex formation. However, in the scientific literature, it has not yet been shown by COP–THB blends can form insoluble hydrogel nanofibers through a treatment. The ability to control the size and nature of the particles, a more persistent drug release profile, and good physical, chemical, and biological stabilities are all advantages of using hydrogel matrix networks as drug carriers (Hoare & Kohane, 2008; Utech & Boccaccini, 2016). Since many of these medications are relatively insoluble in water, which promotes their association with hydrogels, polymer hydrogels have been examined as drug carriers.

The aim of the current study is the improvement and assessment of LDC-loaded COP–THB hydrogels as carrier methods, respectively, for topical delivery of LDC aiming to produce a rapid-acting and long-lasting topical formulation. To examine the aspects that influenced the physical, mechanical, and rheological properties of COP–THB hydrogels, compositions containing different amounts of COP and THB at quantities above the gelation point were characterized. The drug affinity for the COP–THB hydrogels was measured, and the drug release mechanism was examined *in vitro*. Since the COP–THB hydrogels method had low toxicity and were effective in drug delivery, the LDC loaded COP–THB hydrogels dosage forms obtained could provide extended penetration anesthesia without significant toxicity in rats. The preparation and characterization of a new carrier device for the LDC, with formulations in clinical procedures where this anesthetic is commonly used, are significant characteristics of this work.

## 2. Materials and methods

### 2.1. Materials

Polyvinylpyrrolidone (PVP, *M*_w_ = 40,000 and viscosity 2.4 CP), PVA (98–99% hydrolyzed, *M*_W_ = 31,000–50,000), Sodium tetrahydroxyborate decahydrate (borax), lidocaine hydrochloride (LDC) were also obtained from Sigma-Aldrich Ltd. (St. Louis, MO). Dialysis tubing (cellulose membrane) was purchased from Sigma-Aldrich Ltd. with an average flat width of 33 mm and a specified *M*_W_ cutoff of 14,000 Da. All other reagents and solvents were purchased with analytical reagent grade without further purification.

### 2.2. Characterizations

The mechanical properties of the COP–THB hydrogels at room temperature were tested using a tensile tester (INSTRON 3365, Norwood, MA). The specimens were tested with a TA Instruments ARES-LS2 rheometer (TA Instruments, New Castle, DE) equipped with a 50 mm stainless steel upper plate and a 600 grit sandpaper Peltier bottom plate (47185A51, McMaster-Carr, Elmhurst, IL) to prevent slippage to test the rheological behaviors of COP–THB hydrogels. A Shimadzu UV-1601 spectrophotometer at 280 nm analyzed the LDC release content of the solution and reported the data to measure the cumulative release rate (CRR) of the LDC-loaded COP–THB hydrogel system. The morphology of the COP–THB and COP–THB/LDC hydrogels was obtained by scanning electron microscopy (SEM) (S-4700, Hitachi Limited, Tokyo, Japan) using 15 kV for electron beam scanning using a sputter coater after coating with Au. A MFP-3D-Bio (Asylum Testing, San Deigo, CA) with cantilever 'HQ:NSC15/Al-BS' (μMesch) with 40 nN/nm stiffness in AC mode was used to measure the morphology of COP–THB and COP–THB hydrogel loaded with LDC (tapping mode).

### 2.3. Fabrication of COP–THB hydrogel

PVP/PVA (COP) (20/10% w/w; 1:1 ratio) and THB (8% w/w; 0.2, 0.4, 0.6, 0.8, 1.0, and 1.5 ratio) stock solutions were prepared in deionized water (Husain et al., 2018; Al-Emam et al., 2020). Hydrogels were developed to create a fluid gel by combining the required ratios of both formulations for approximately 1 h with frequent stirring. Gels were stored for 48 h at room temperature sealed poly(propylene) containers (44 mm diameter, 55 mm depth) to allow complete gelation before evaluation. To get the mixture back to its original weight, adjustments for any weight loss were adjusted by the addition of deionized water. Until further study, to allow complete gelation and removal of air trapped. LDC-loaded COP–THB hydrogels were prepared by the method described above (Ma et al., 2017), but with minor modification. COP and THB concentrations in the final hydrogel were adjusted to a 1.0:1.0 weight ratio. Sufficient LDC was added to produce loadings of 0.5, 1.0, and 2.0% w/w in the final formulation.

### 2.4. *In vitro* cytotoxicity assay

In DMEM, supplemented with 10% FBS and 10 units/mL penicillin–streptomycin sulfate at pH 7.4, the cytotoxicity of COP–THB and COP–THB/LDC composite hydrogels was tested using mouse fibroblast L929 cells ( Wang et al., 2021). The cells were seeded with a density of 5 × 10^4^ cells/well on 96-well plates. Reduction of MTT assay was tested for cell viability. Then the before exposure to COP–THB and COP–THB/LDC for 24 h. MTT solution (10 μL) was added to each well and cells were incubated for 1 h at 37 °C. The MTT assay was performed at extended periods of 24, 48, and 72 h. Dose–response curves were plotted to determine the half-maximal inhibitory concentration (IC_50_) for COP–THB hydrogels in the presence and absence of LDC. The relative absorbance at 490 nm was measured using a Varioskan Flash microplate reader (Thermo Scientific, Waltham, MA) of the treated cells versus the control (untreated) cells was used in calculating the percentage cytotoxicity and percentage viability. Percentage cytotoxicity was expressed as IC_50_. Images were taken on an IX81 Olympus inverted microscope (Olympus, Tokyo, Japan):

% Cytotoxicity = Abs of control – Abs of sample/Abs of control × 100

% Viability = 100 − % Cytotoxicity

### 2.5. Animals

All experiments were carried out using 5-week old, male IGS (ICR) mice purchased from the Zhengzhou University Medical Animal Test Center (China). The rats were maintained in the experimental animal facilities at the Zhengzhou University. The rats were maintained under controlled conditions (temperature, 20 ± 1 °C; humidity, 60 ± 5%; 12/12 h light/dark cycle) and fed commercial chow and water ad libitum. On the day preceding the experiment, the abdominal hair was shaved. The Ethics Committee of Zhengzhou University has accepted and ratified all animal experiments.

### 2.6. *In vivo* evaluation of anesthetic effect in rats

Local anesthetic effects of LDC covering formulations were assessed by humans and animals as a way of determining anesthetic/analgesic effects (Zhang et al., 2017; Sawai et al., 2019; Fukunaga et al., 2020). Animals were divided into separate experimental groups at random, and 50 mg of different doses were introduced to the midline of the tail in a 2.0–2.5 cm region. In brief, the injection into the hind paws of rats of 100 μL of COP–THB/LDC (15 mg/mL of LDC in PBS (pH = 7.4) containing 35 mg/mL of COP–THB hydrogels) equivalent to 200 mg/kg body weight rats for COP–THB hydrogels and 50 mg/kg body weight for LDC after 16 h. To cause inflammation, 100 μL of carrageenan (CGN) solution (0.20 wt% in PBS) was injected into the same site. Since the pain quickly decreased after treatment of CGN, we used this experimental procedure. With Von Frey hair (VFH, Stoelting, IL) with a bending force of 25 g, the injection sites were labeled and tested six times. Each rat was shaved with an area of 4 × 5 cm on its back skin. Each labeled area was subcutaneously injected with 0.1 ml of medication and the reaction of the rats to the injection was checked after 15 min. The skin at the injection sites was harvested at 1 and 5 days post-injection and the hydrogels remaining at the injection sites were excised to permit visual inspection. The mean of three separate measurements taken at 10-min intervals was used to determine the prodrug latency. To prevent tissue damage in analgesic animals, a maximum cutoff latency of 10 s was chosen.

### 2.7. Histopathological evaluation

After 24 h, mice treated with RIG and control groups were sacrificed. Mice hind paws were picked, fixed in 10% neutral buffered formalin, coated in paraffin, sectioned (5 m thick), and stained with hematoxylin and eosin (H&E) and Masson's trichrome stain (MTS) (Barthel et al., 2003). Usage of the Keyence BZ-X710 microscope to analyze tissue parts (Keyence, Co., Tokyo, Japan). Rats were rasped on the back and split into three classes at random: COP–THB/LDC, COP–THB, and blank PBS. Samples (0.3 mL) were injected into rats subcutaneously. In addition, rats were also extracted and tested for any anomalies and detectable liver toxicities from the respective treatment groups.

### 2.8. *In vivo* toxicity study

Two rats (200 and 250 g) were injected subcutaneously through the tail vein with LDC-loaded COP–THB at a final formulation (the dose was 2 mg/kg in 1 mL phosphate-buffered saline) once every 4 days for a total of four times. Two rats were used as controls without injection. The rats were then dissected and tissues from the kidney, liver, and spleen were removed for histopathological analyses (Wang et al., 2016). The tissues were then washed in xylene before being impregnated twice with molten paraffin wax. All mice were monitored by the tumor volume (V) was calculated according to the formula: *V* = 4*π*/3 × (tumor length/2) × (tumor width/2)^2^. After 21 days, the mice in each group were sacrificed and the organs and blood sample were separated from the bodies to measure the H&E staining and blood biochemical index. The samples were embedded in paraffin for hematoxylin and eosin (H&E).

### 2.9. Statistical analysis

All studies were repeated at least three times and reported as means ± standard deviation. To assess systematically important variations, a one-way analysis of variance was used, followed by Tukey's multiple comparison test. Results with a significance level of *p* .05 were considered statistically significant.

## 3. Results and discussion

### 3.1. Mechanical properties of COP–THB hydrogel

**Figure 1.**
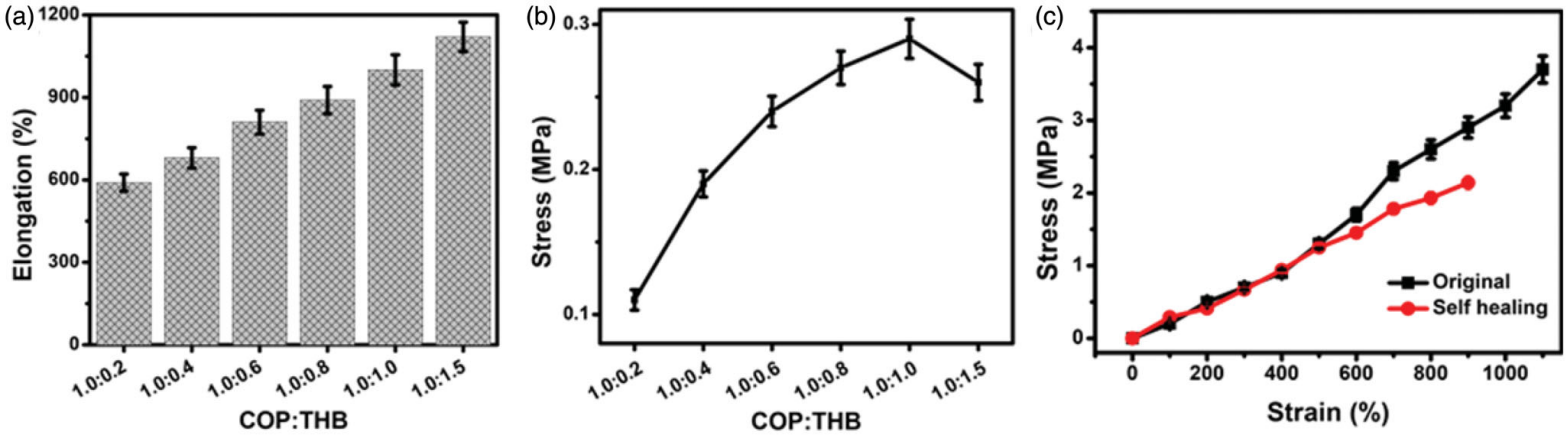
(a) COP–THB hydrogels under stress–strain mechanical test. (a) Elongation of the COP–THB hydrogels (b). (a) Stress of the COP–THB hydrogels and (c) stress–strain curves of original hydrogel and self-healed hydrogel with COP:THB of 1.0:1.0.

The developed materials were prepared to ensure that they experience elastic deformation when subjected to compressive load (Silva et al., 2019). Therefore, it is important to determine the elastic-to-plastic transition, as well as the energy required up to rupture. To study the mechanical properties of the COP–THB hydrogels, stress–strain mechanical tests of hydrogels with different COP:THB ratios were carried out. To test its mechanical properties, the COP:THB hydrogels = 1.0:0.1 is too soft (Yiyin Zhang et al., 2019). The tension of the hydrogels significantly increased as the quantities of THB increased, as shown in Figure 1(a,b). As the THB increased to 1.0, the elongation reached 1000 ± 55% of the original length and the stress was 0.29 ± 0.01 MPa, indicating strong mechanical properties for the hydrogel of COP:THB = 1.0:1.0 (Zhu et al., 2017). To further measure the hydrogel in this experiment had higher mechanical properties performance of COP:THB = 1.0:1.0 than some bio-based COP:THB = 1.0:1.5 ratio of hydrogels. The compact network structure can be blamed for the rise in hydrogel tension. The self-healed hydrogel of COP:THB = 1.0:1.0 was prepared by cutting the samples in half with a blade and re-contacting without even any external stimulation to more analyzing the mechanical characteristics of the healed hydrogel (Ren et al., 2020). It demonstrates that the concentration of THB for crosslinking also affects the mechanical properties of COP–THB hydrogels. However, after the introduction of THB (1.5 ratio), the mechanical properties of composite hydrogels decreased, which was possibly due to the comparatively larger particle size of THB and the lack of contact between THB and COP networks. Figure 1(c) reveals the initial and self-healed hydrogel's standard strain–stress curves. The pressure and tension of self-healing were 1027 ± 84% and 2.14 ± 0.10 MPa. These findings indicate that, largely after injury, the healed hydrogels can recover their structure and COP–THB hydrogels have strong self-healing properties (Deng et al., 2020). We can conclude that the particles are literally dispersed within the hydrogel network in this case, and that particle agglomerates are also integrated into the bulk of the hydrogel.

### 3.2. Rheological properties of COP–THB hydrogels

**Figure 2.**
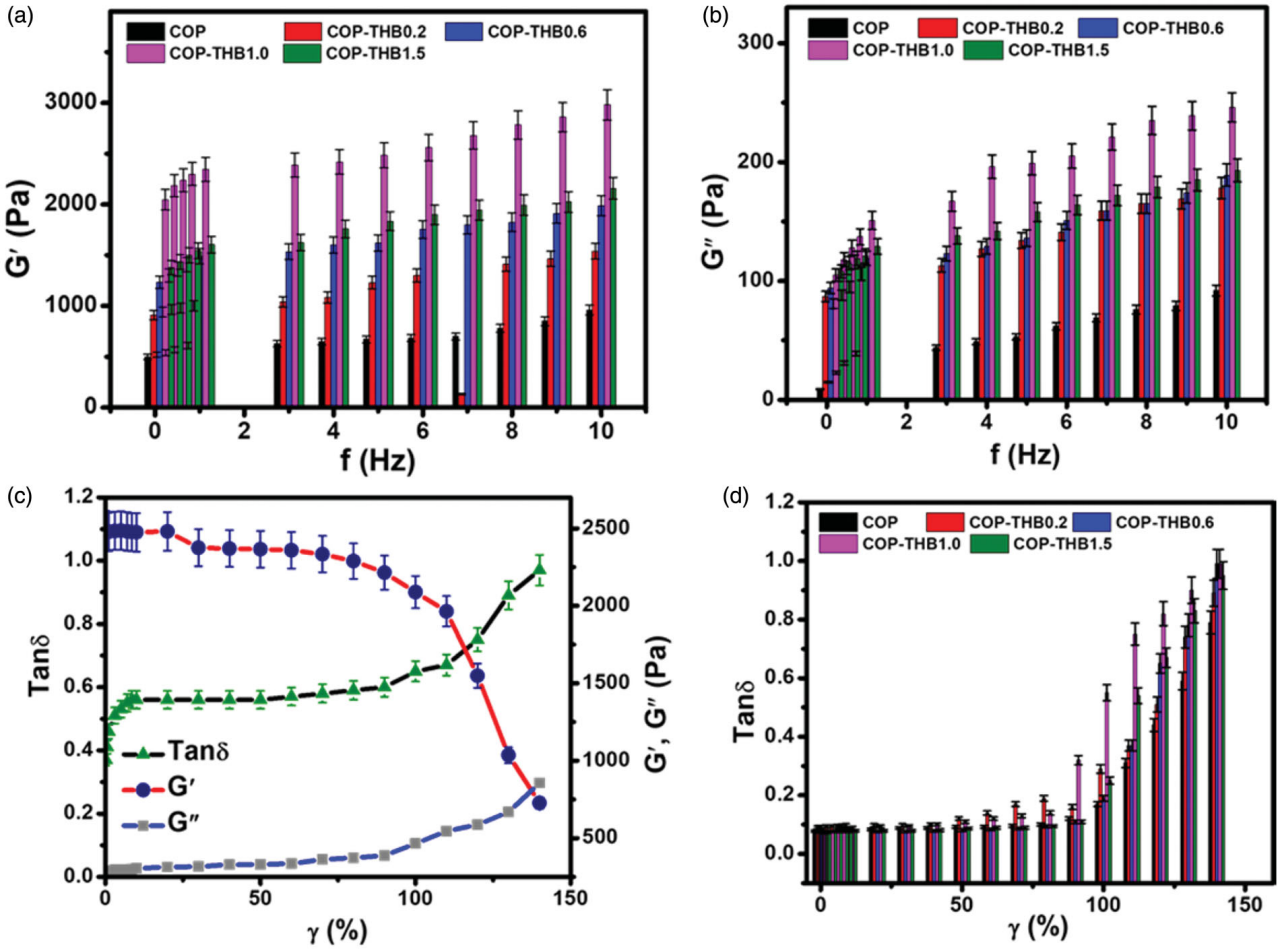
The rheological properties of the COP–THB hydrogels at different frequencies (a and b) and strains (c and d).

The self-healable properties of the chemical cross-linked hydrogels were investigated by multi-step oscillatory rheological measurements. To well investigate the rheological properties of COP–THB hydrogels, rheological tests at various ratios were also performed. The rheological behavior of COP–THB hydrogels was investigated, and results are shown in Figure 2. As shown in Figure 2(a,b), different results were also achieved, indicating the solid-state of the COP–THB hydrogels with ratios rising, both higher storage modulus (G') than the loss module (G"). At the same time, the COP–THB hydrogels improve both G' and G" values along with the frequency, which also proves that the COP–THB hydrogels are constructed of complex Schiff base bond and hydrogen bond systems (Xu et al., 2018). When the concentration of THB was added, the gelation was significantly faster than those of other ratios of hydrogels. Additionally, COP–THB(1:0) hydrogels have higher G′ and G″ values than those of COP–THB(1:5) hydrogel illustrate the maximum G′ and G″ values over others. There were homogeneous porous structures, with some large holes spread between small holes, which may be due to changes in ratios. Rheology research has shown that the intermolecular bonds between COP and THB are strongly broken, decreasing the number of intermolecular bonds and creating large pores in the structure (Ali & Shah, 2021). The strain dependence of G′, G′′, and the loss factor tan δ of the COP–THB(1:0) hydrogel is shown in Figure 2(c,d). G′, G′′, and tan *δ* of the COP–THB hydrogel remain stable in the small strain field, indicating a linear viscoelastic state. When the pressure is high enough, G′ starts to fall sharply, while G′′ and tan δ both rises at the same time. This is expressed by the rise in tan *δ* values (tan *δ* > 1) when the application of frequency is intensified. For COP–THB(1:0) hydrogel, this pattern is also observed, but for the whole frequency spectrum, this remains solid-like. Notably, the COP–THB(1:0) composition could rapidly shape hydrogels even before the rheology test, as demonstrated by the larger G′ than G′′ in the study.

### 3.3. Water content and swelling ratio of COP–THB hydrogel

**Figure 3.**
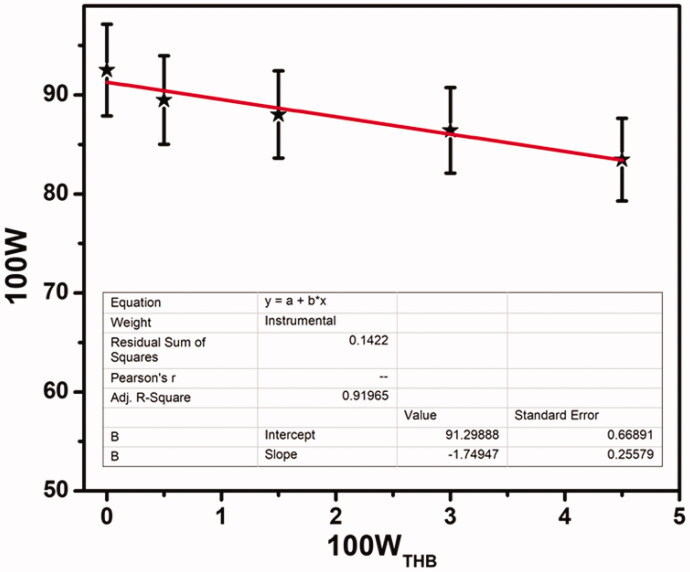
The dependence of the water content (100 W, mass percent fraction) on the mass percent fraction of THB (100W_THB_); the standard deviation is shown in bars. The average standard deviation is 0.05%.

**Figure 4.**
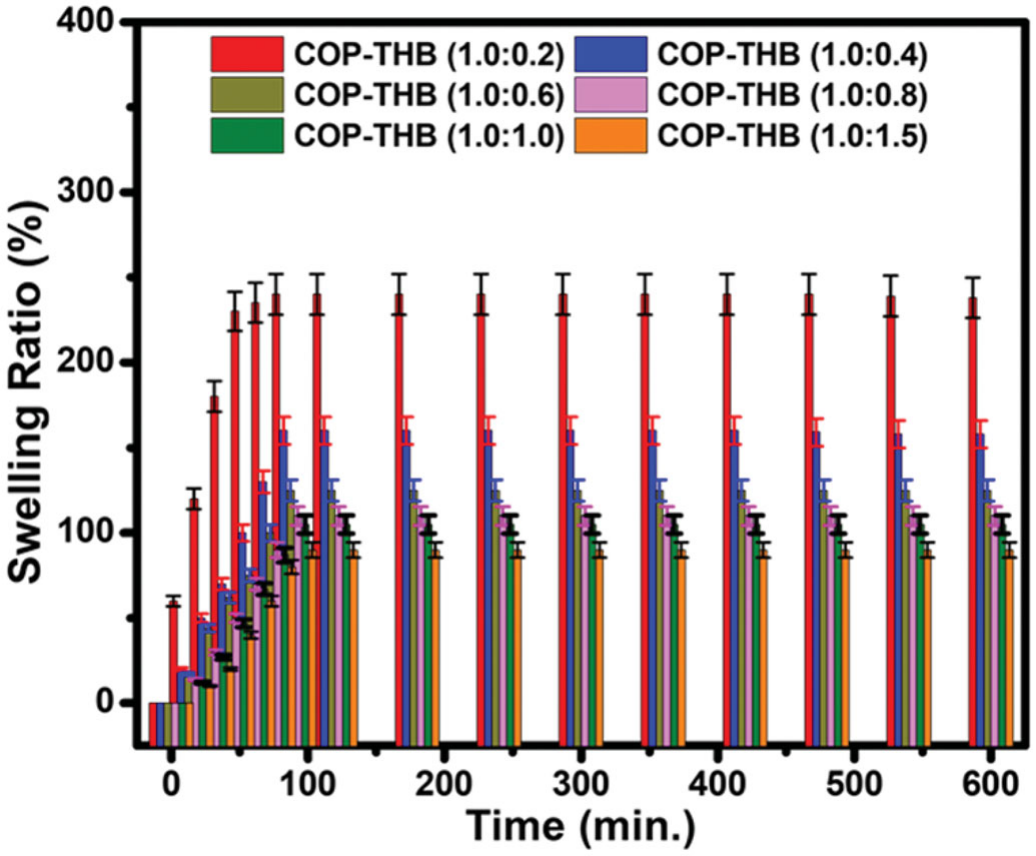
The swelling ratio of various ratios of COP–THB hydrogels as a function of time.

High water content (up to 90% water) is achieved for the dual crosslinking hydrogel (Li et al., 2015). As we know, water content is a basic property of hydrogels. The water content needed for use in local anesthetics is higher. In addition, the content of water represents the free space of molecular motion and is relative to the structure of the hydrogel network. The intensity of the hydrogel decreases as the volume of water increases (Kudo et al., 2014). Figure 3 shows the dependence of water content (W, mass fraction) on the mass fraction of THB (W_THB_, mass fraction) for COP–THB hydrogels. A linear decrease relationship between the W and the W_THB_ is observed. For hydrogels, the network space is occupied by water. As we expected, the water content of the COP–THB hydrogels was further increased when they were allowed to swell in water to equilibrium. The high-water content of COP–THB hydrogel can benefit significantly from the hydrophilicity in the hydrogel, such as the hydroxyl, carboxyl, and amido groups, contributing to the high hydration ability and leading to a high-water content, although this study does not further explore the mechanism (Khunmanee et al., 2017). The water content of the COP–THB hydrogels at 10:1.0 water:polymer ratio at equilibrium was 92.28 ± 4.6% water content by mass and minimum value of 83.54 ± 4.1%. To the best of our knowledge, there were no reports on hydrogel with a water content of more than 90%. However, as the water content of the hydrogels increased, the mechanical properties of the hydrogels deteriorated significantly. Hydrogels with a higher water content are more sensitive and excellent biocompatibility, making them better suited for biological applications (Jiang et al., 2020). One of the most essential factors to consider when measuring COP–THB hydrogels is the swelling ratio. Swelling analysis was carried out under various conditions to find the impact of THB on the swelling activity of COP–THB hydrogels. First, the relationship between swelling ratio and time was investigated. Figure 4 demonstrates the hydrogels swelling behavior after many hours of immersion in water. The final swelling ratio exceeds 242 ± 12% when the ratio of COP and THB is 1.0:0.2. Since THB occupies space in the matrix throughout the synthesis of THB loaded hydrogels, and when submerged in water, this occupied space results in slightly more swelling compared to unloaded hydrogels (Budianto & Amalia, 2020). COP:THB-1.0:1.5 exhibits superior swelling property that the SR value is 90 ± 4.5% in the saturated state. The free THB molecules can interact with the cross-linked THB molecules as THB concentration increases in the medium and break the COP:THB-1.0:1.0 cross-link. This is due to the presence of a large number of hydrophilic hydroxyl groups on the polymer backbone, leading to the enhanced hydrophilic properties of the hydrogels.

### 3.4. Drug release of COP–THB/LDC hydrogels

**Figure 5.**
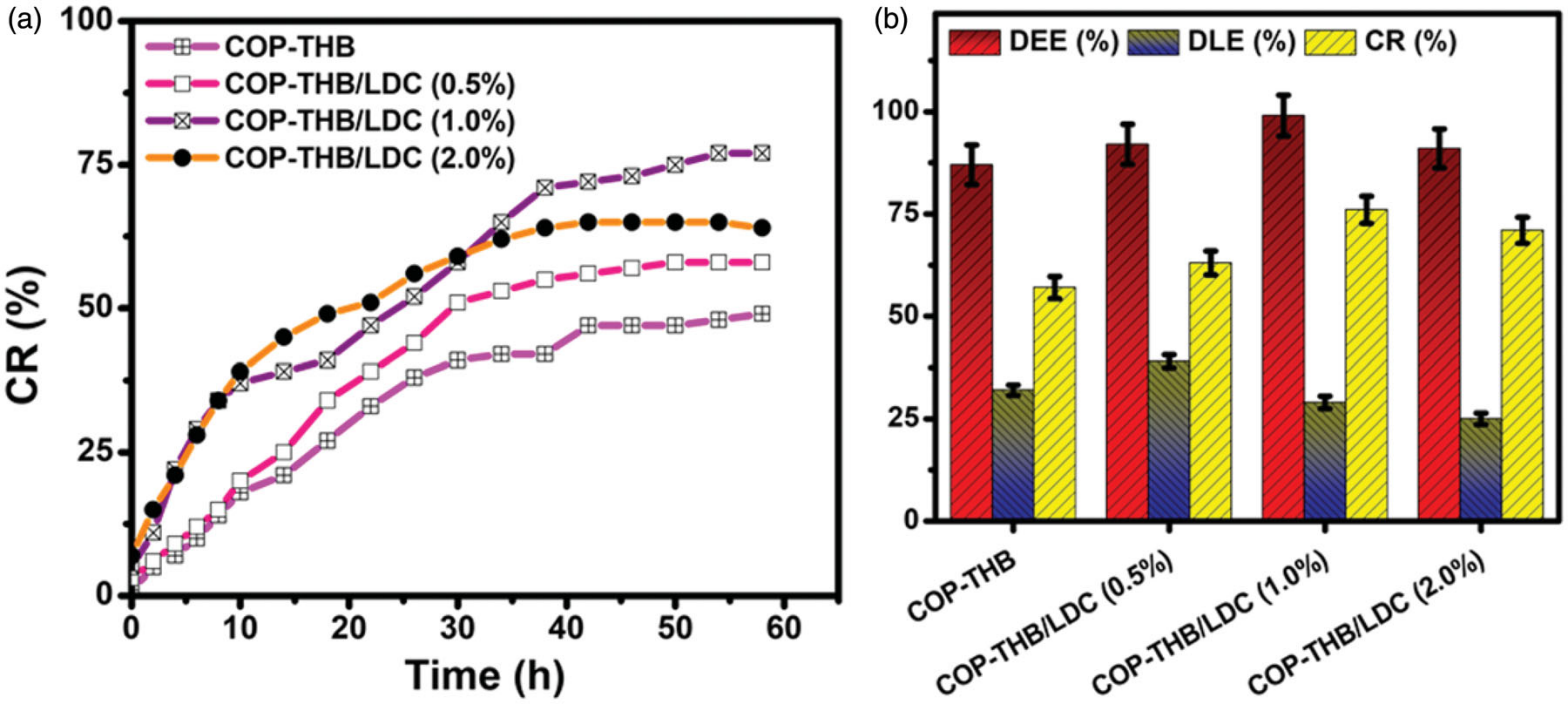
The drug sustained-release curve (a) and data (b) of encapsulation efficiency (%, DEE), drug loading efficiency (%, DLE), and cumulative release rate (%, CCR) of COP–THB/LDC hydrogels in 37 °C PBS solution.

Understanding how drugs are released from drug-carrying hydrogel-based lenses will help with the creation of more effective drug delivery systems. The drug release of COP–THB hydrogels showed a different release profile as shown in Figure 5. LDC was also rapidly released from the base retention of COP–THB/LDC hydrogels, but more slowly than from LDC in all tested conditions, with the total release of COP–THB/LDC hydrogels. For example, the drug release reduces from approximately 76.5 ± 0.9% in 36 h to reach equilibrium (Lee & Kim, 1991). Figure 5(a), the range from 58.7 ± 1.2 to 66.2 ± 0.9% in the 56 h after loading with COP–THB/LDC hydrogels, which indicates that the copolymer can effectively control burst drug release and achieve sustained drug release. The increase in the number of hydrophilic groups in the blends helped to increase intramolecular H-bonding, which helped to include the drug (Alhosseini et al., 2012; Abasalizadeh et al., 2020). COP–THB/LDC hydrogels drug loading rate ranges from 25.6 ± 0.5 to 32.5 ± 0.9%, which is also higher than that of COP–THB hydrogels. The drug is released from the combined hydrogels when water diffuses through the polymeric network because it is water-soluble. This causes the hydrogel to swell and the substance to dissolve in the solution. Several standard mathematical equations were equipped with the release kinetics of LDC to illustrate the release mechanisms of LDC from COP–THB/LDC hydrogels (Paolino et al., 2019). For all of the versions, the *R*^2^ was > 0.9. The Higuchi model showed the best fit for the data, indicating that hydrogels from COP–THB/LDC give a monitored or release profile of LDC. *In vitro* drug-release kinetic model of COP–THB/LDC fit well with the Higuchi equation: *Q* = 2.64*t*_1/2_ + 1.37 (*R*^2^ = 0.9963) compared with the other methods such as zero-order equation: *Q* = 2.03 + 1.02 (*R*^2^ = 0.9495), first-order equation: ln(1−*Q*) = 2.37*t* − 0.97 (*R*^2^ = 0.9238), and Weibull’s equation: ln[1/(1−*Q*)] = −1.36ln*t* + 1.68 (*R*^2^ = 0.9728).

### 3.5. Cytotoxicity tests of COP–THB/LDC hydrogels

**Figure 6.**
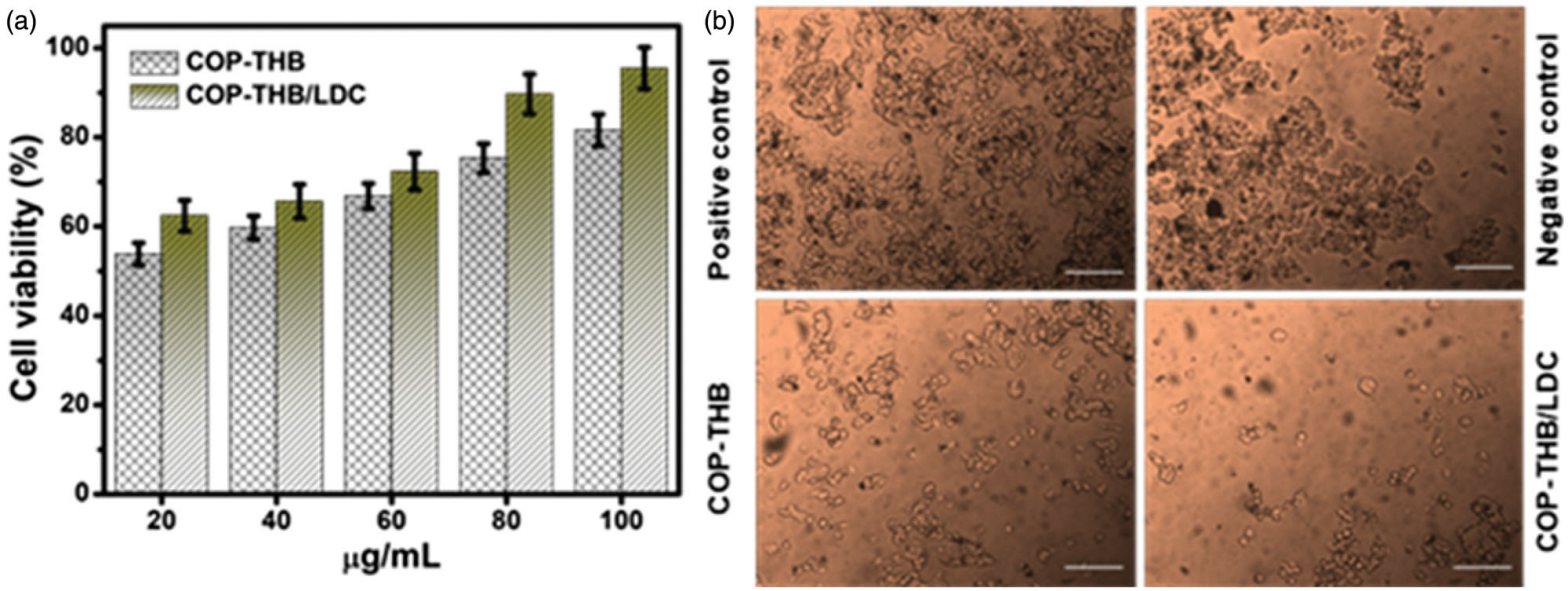
(a) Cell viability (MTT assay) of mouse fibroblast L929 cells after 24 h exposure to various concentrations of COP–THB and COP–THB/LDC hydrogels ranging from 20 to 100 μg/mL. (b) Images of L929 cells under an inverted microscope in positive and negative control and in presence of 100 μg/mL COP–THB and COP–THB/LDC hydrogels. The scale bar at 100 μm.

To assess the cell cytotoxicity of COP–THB and COP–THB/LDC hydrogels, mouse fibroblast L929 cells were used *in vitro* MTT assay to evaluate the cell viability of the hydrogels. Cytotoxic activity was evaluated at the concentration of COP–THB and COP–THB/LDC hydrogels ranging from 20 to 100 μg/mL (Figure 6(a)). A significant increase in cell viability was observed when the cells were incubated COP–THB hydrogels (20 and 100 μg/mL) mixed with LDC after the treatment for 24, 48, and 72 h. The viability of L929 cells treated with the 20 μg/mL of COP–THB hydrogels and COP–THB/LDC for 24 h was 53.8 ± 2.49% and 62.4 ± 3.47%, whereas the cell viability of cells treated with 100 μg/mL of COP–THB hydrogels and COP–THB/LDC were 81.6 ± 3.58 and 95.5 ± 4.67%, respectively (Zhang et al., 2017). After 48 h and 72 h of incubation, % cell viability of L929 cells treated with 100 μg/mL of COP–THB hydrogels and COP–THB/LDC decreased to 69.7 ± 3.48 and 75.2 ± 3.76% for 48 h; 41.9 ± 2.09 and 56.8 ± 2.84% for 72 h, respectively (Figure S1). This implies that living cells are not affected by the presence of LDC and COP–THB/LDC hydrogels are non-cytotoxic (Weng et al., 2008). This suggests that the stimulation by COP–THB/LDC hydrogels of cell viability was dosage and time-dependent. A significant difference in the IC_50_ value between free COP–THB hydrogels and COP–THB/LDC formulations was observed. The strong binding of the COP–THB hydrogels on the surface of LDC, as indicated by the in vitro release, resulted in similar cytotoxicity between free COP–THB hydrogels and COP–THB/LDC (IC_50_ of 21.4 ± 1.3 versus 47.3 ± 3.9 μM for free COP–THB hydrogels and COP–THB/LDC, respectively). However, > 2.5-fold increase in the IC_50_ value of the LDC drug was observed when it was complexed with COP–THB/LDC (IC_50_ of 47.3 ± 3.9 μM) reflecting the slow release of LDC from the COP–THB hydrogel to the cells. It can be inferred, based on the above data, that COP–THB/LDC hydrogels are possibly beneficial alternative biomaterials for tissue engineering applications. After incubation, cultures were studied under an inverted microscope for changes in cancer cell morphology caused by the cytotoxic effects of COP–THB and COP–THB/LDC hydrogels. The numerous morphological variations after treatment with 100 μg/mL concentrations of hydrogels are shown in Figure 6(b). The cells shrank in size, and the cytoplasm became compact and more tightly packed.

### 3.6. Microscopy analysis of COP–THB/LDC hydrogels

**Figure 7.**
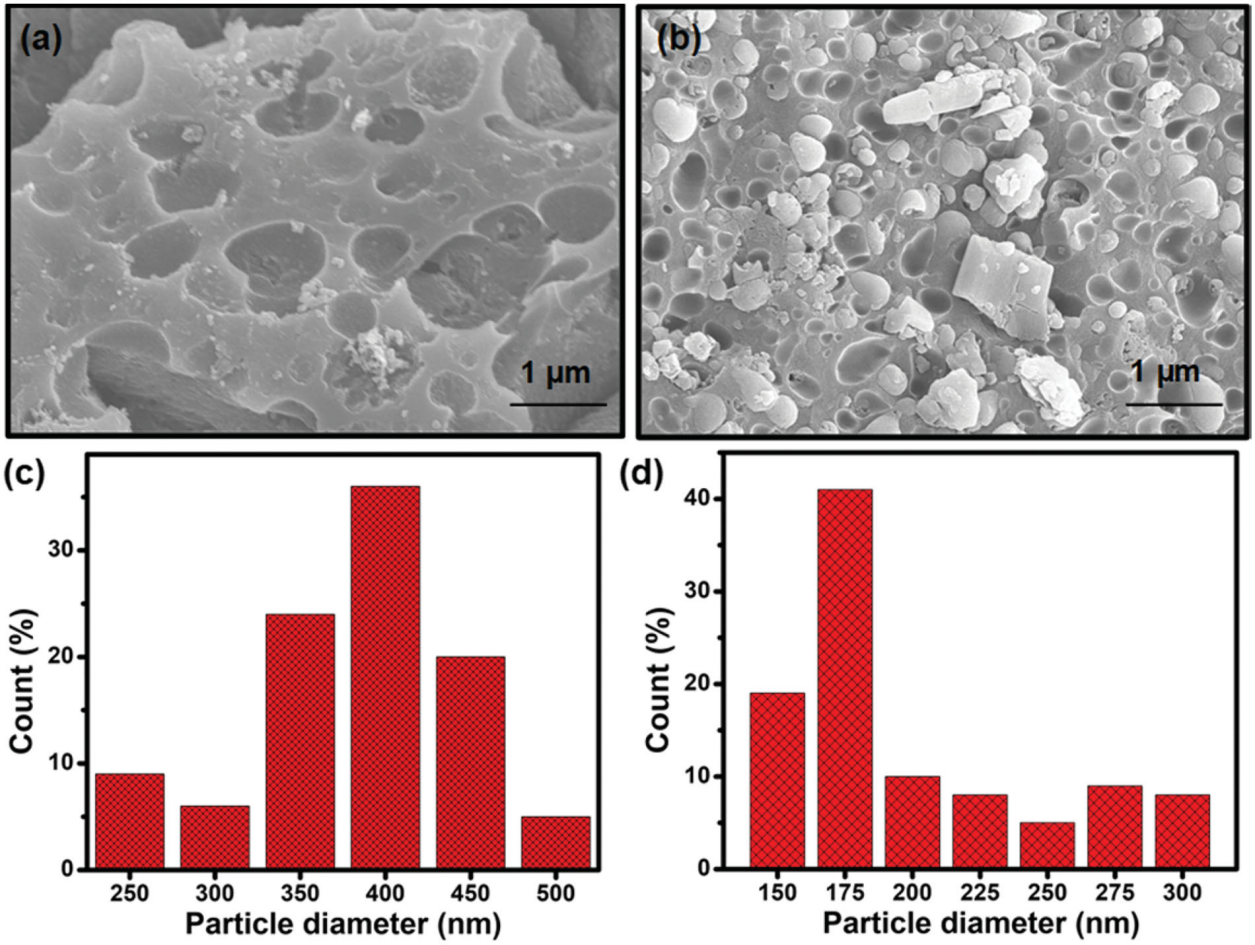
SEM image of (a) COP–THB hydrogel and (b) COP–THB/LDC hydrogels composites. The average pore size of (c) COP–THB and (d) COP–THB/LDC hydrogels composites.

The scanning electron microscopy (SEM) was used to investigate the surface porous morphology of COP–THB and COP–THB/LDC hydrogels as shown in Figure 7. Figure 7(a,b) shows the SEM images of scaffolds freeze-dried from hydrogels. Frozen water in the polymer structure quickly sublimed, leaving voids or pores without altering the structure of the hydrogel (Kaberova et al., 2020). It can be found that a porous and cross-linked structure displays COP–THB and COP–THB/LDC hydrogel. In addition, COP–THB and COP–THB/LDC hydrogel interactions and formation are measurable and the pore size with different diameter ranges indicates the existence of porosity of the hydrogels. Apparently, from Figure 7(a,b), the pore size decreases of COP–THB hydrogels with the addition of LDC. This phenomenon can be attributed to the fact that the network space filled with LDC, and the interaction between LDC and COP–THB lead the hydrogels space to be compressed. The surface morphology of spherical COP–THB and COP–THB/LDC hydrogel when analyzed at higher resolution exhibited a highly interconnected porous structure with an average pore size of 395 and 182 μm (Figure 7(c,d)) (Hou et al., 2020). Many studies have found that porous structures with a diameter of 150–300 mm are useful for tissue regeneration (Loh & Choong, 2013; Bružauskaitė et al., 2016). The pore size range of the COP–THB/LDC hydrogels was 150–250 mm, which is ideal for biomedical applications. To allow adequate nutrients and cell growth, an ideal scaffold for tissue-engineered should have at least 90% porosity.

**Figure 8.**
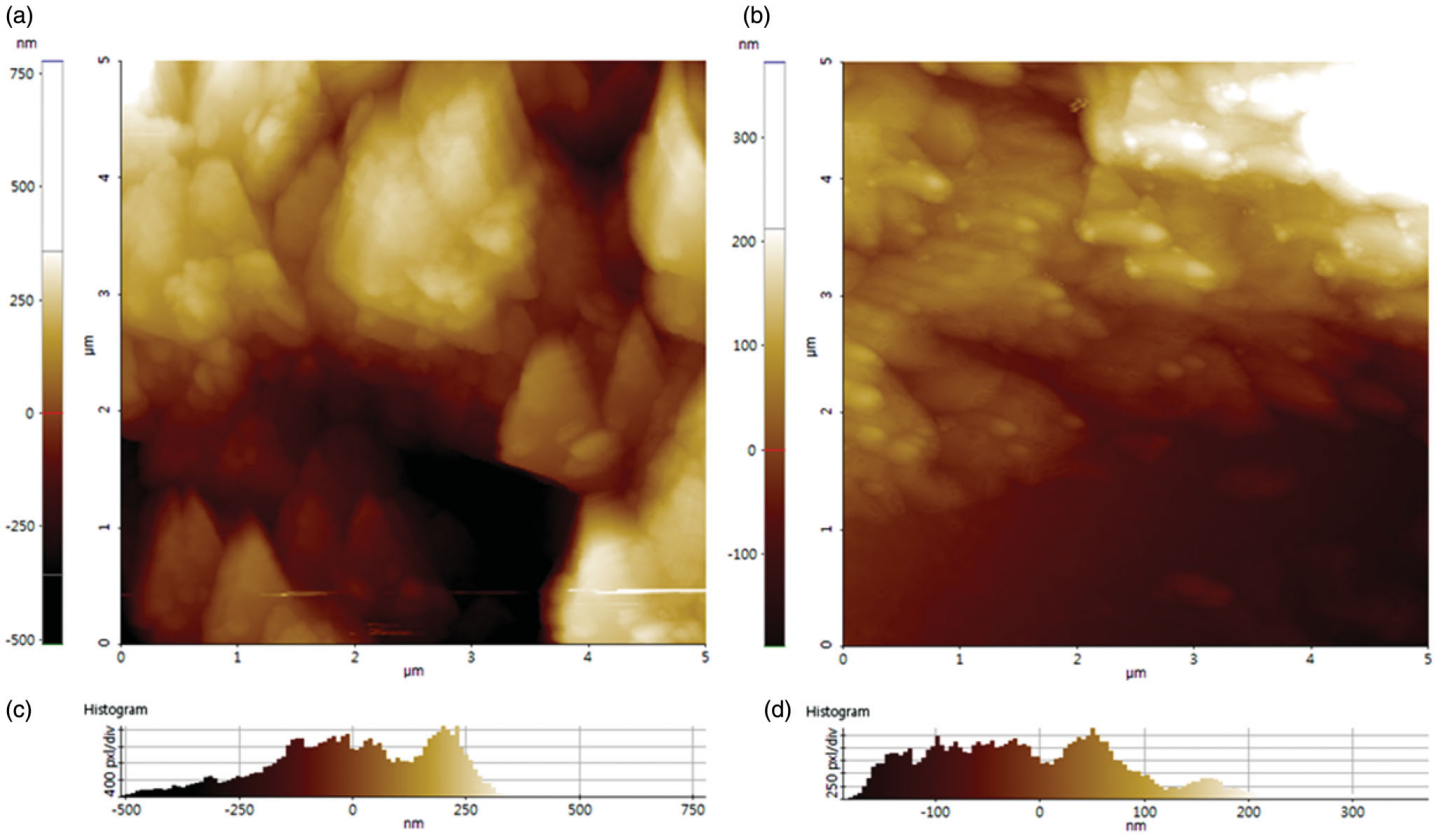
AFM image of (a) COP–THB hydrogel and (b) COP–THB/LDC hydrogels composites. Height histogram profile of (c) COP–THB and (d) COP–THB/LDC hydrogels composites.

The surface morphology of COP–THB and COP–THB/LDC composite hydrogel was also investigated using the AFM technique as shown in Figure 8. The particles of COP–THB and COP–THB/LDC hydrogels were found to be spherical, with diameters ranging up to 500 nm, where some portions are essentially noted to be provided by the interconnected pore walls of hybrid hydrogel (Figure 8(a,b)). The diameters of COP–THB and COP–THB/LDC hydrogels determined by AFM were smaller than the sizes of COP–THB/LDC (∼200 nm) hydrogels obtained from COP–THB (∼500 nm) (Lee et al., 2016). The histogram results in Figure 8(c,d) show that LDC has a homogeneous distribution COP–THB in the nanocomposite matrix with an average size up to 500 nm. As a result of the strong interaction between LDC and COP–THB hydrogels solution along with the destruction of the cross-linking capacity of the hydrogels matrix, this increase in surface roughness of COP–THB/LDC hydrogels can be allocated as a highly porous structure (Parhi, 2017). The average roughness of the polymer sample affects the drug release mechanism. As confirmed by the inset 2D image of COP–THB and COP–THB/LDC hydrogels, the dark region (pore walls) is observed to be diminished by the appearance of the yellow-red region, indicating the decrease in pore dimension. Cell adhesion, proliferation, and differentiation of cells in the tissue are all assisted by the rough surface of the polymer films used for tissue treatment. In conjunction with the above-mentioned SEM and AFM analysis further confirmed that the polymer with tailored structure was successfully synthesized, which were then used as a cross-linker for the synthesis of COP–THB/LDC composite hydrogel.

### 3.7. Anesthetic effect of COP–THB/LDC

**Figure 9.**
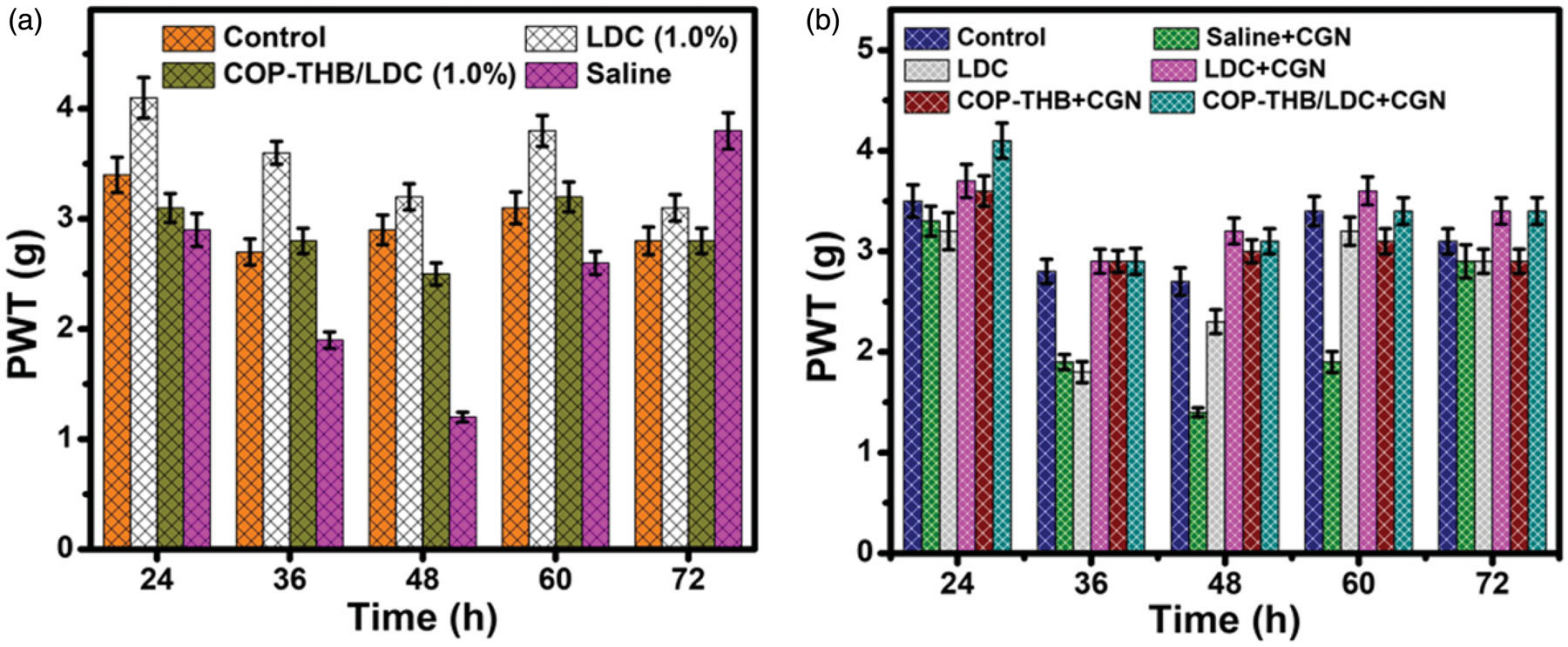
The local anesthetic effect of COP–THB/LDC complex in the absence (a) in the presence (b) of CGN. All rats were injected 50 μL of the sample. The hind paw withdrawal thresholds (PWT, (g)) are shown as mean ± SEM (*n* = 5 in each group). *p < .05 compared with the control and COP–THB/LDC group.

In our study, rats treated with saline or blank LDC shivered in response to each prick, indicating that the COP–THB itself did not cause local anesthetic effects. Animals treated with COP–THB/LDC showed rapid onset of local anesthesia. After we confirmed that COP–THB/LDC has a longer retention time than COP–THB solution, we investigated the local anesthetic effect of COP–THB/LDC *in vivo* (Abendschön et al., 2020). We used carrageenan (CGN)-induced inflammation on the RHP. The *in vivo* antinociceptive effect (sciatic nerve block) was evaluated through the paw withdrawal threshold (PWT) test, a well-described technique to study the anesthetic efficiency in rats (Thalhammer et al., 1995; Villarinho et al., 2013). As illustrated in Figure 9(a), few hours after injection of CGN into the paw, PWT decreased. The tactile sensation started to recover after 1 h in animals treated with COP–THB/LDC solution and anesthesia persisted approximately 10 h; tactile sensation began to recover at approximately 6 h in animals treated with COP–THB/LDC, and anesthesia lasted more than 36 h, which was 6 times longer than with COP–THB solution. It is important that in COP–THB/LDC, the PWT significantly decreased and recovered in 3 days (Xie et al., 2015). However, about 6 h after injection, the effect began to fade as the blood concentration fell, and the rats stinging reaction increased, approaching that of the control group. As a result, selecting a suitable polymer for use as a matrix for controlled drug release is important. Polymers that can reduce patient pain by preventing allergic reactions and extending the therapeutic effect are very useful.

Then, using CGN-induced local inflammation in RHP, we tested the anesthetic effect of COP–THB/LDC. As shown in Figure 9(b), with LDC alone and with a combination of LDC and COP–THB, which was presumably due to the rapid diffusion of COP–THB, the CGN-induced decrease in PWT did not recover. In addition, COP–THB/LDC injection aggravated local inflammation and greatly reduced PWT for a long time. On the contrary, LDC was able to slowly and for a long time release into the body in the COP–THB/LDC hydrogel group due to the time release of the hydrogel (Jacob et al., 2021). Thus, for a longer time, local anesthesia could last. Furthermore, on day 3 after injection, the residual LDC content in the gel at the injection site was found to be 26.5% of the initial level. This suggests that within the first 72 h after injection, COP–THB/LDC will release most drugs and that the residual drug is likely to be harmful to the body. This result is consistent with the evaluation of *in vivo* anesthesia efficacy, which showed that LDC anesthesia lasting 2–3 days is given by our COP–THB/LDC formulation. This exciting finding suggests that by lowering the anesthetic dosage while maintaining the same anesthesia level, much less medication may be used in the clinic, reducing toxicity (El-Boghdadly et al., 2018). These findings showed that the regulated release of anesthetic drugs from COP–THB combined with the hydrogel matrix's ability to scavenge ROS decreased local pain and extended the duration of action *in vivo*.

### 3.8. COP–THB/LDC reduced inflammation in RHP

**Figure 10.**
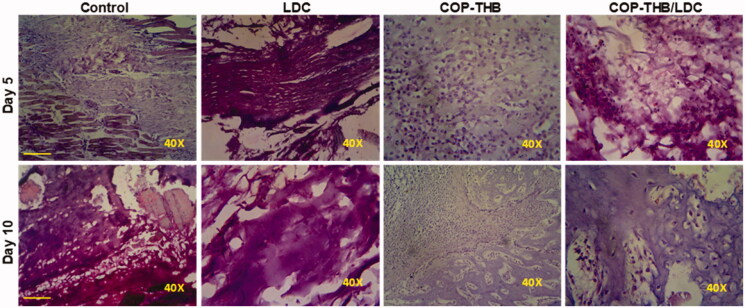
Histopathological evaluation of the anti-inflammatory effects of COP–THB/LDC against CGN-induced inflammation on days 5 and 10. Paraffin sections of rats hind paw were stained with hematoxylin and eosin (H&E) (scale bar = 100 μm).

**Figure 11.**
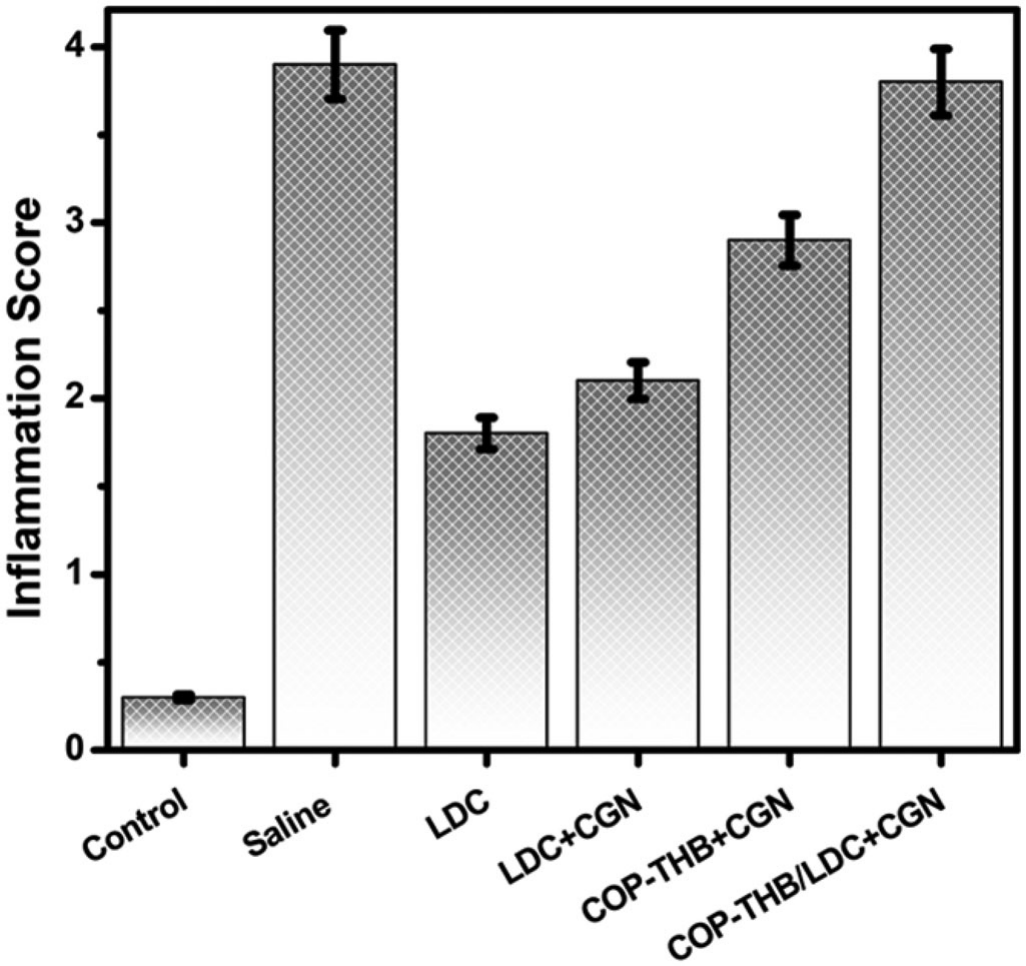
The degree of inflammation was evaluated on a scale of 0–5. Values are expressed as mean ± standard error of the mean. *p* < .05 versus the saline group (CGN-induced inflammation model injected with saline solution).

**Figure 12.**
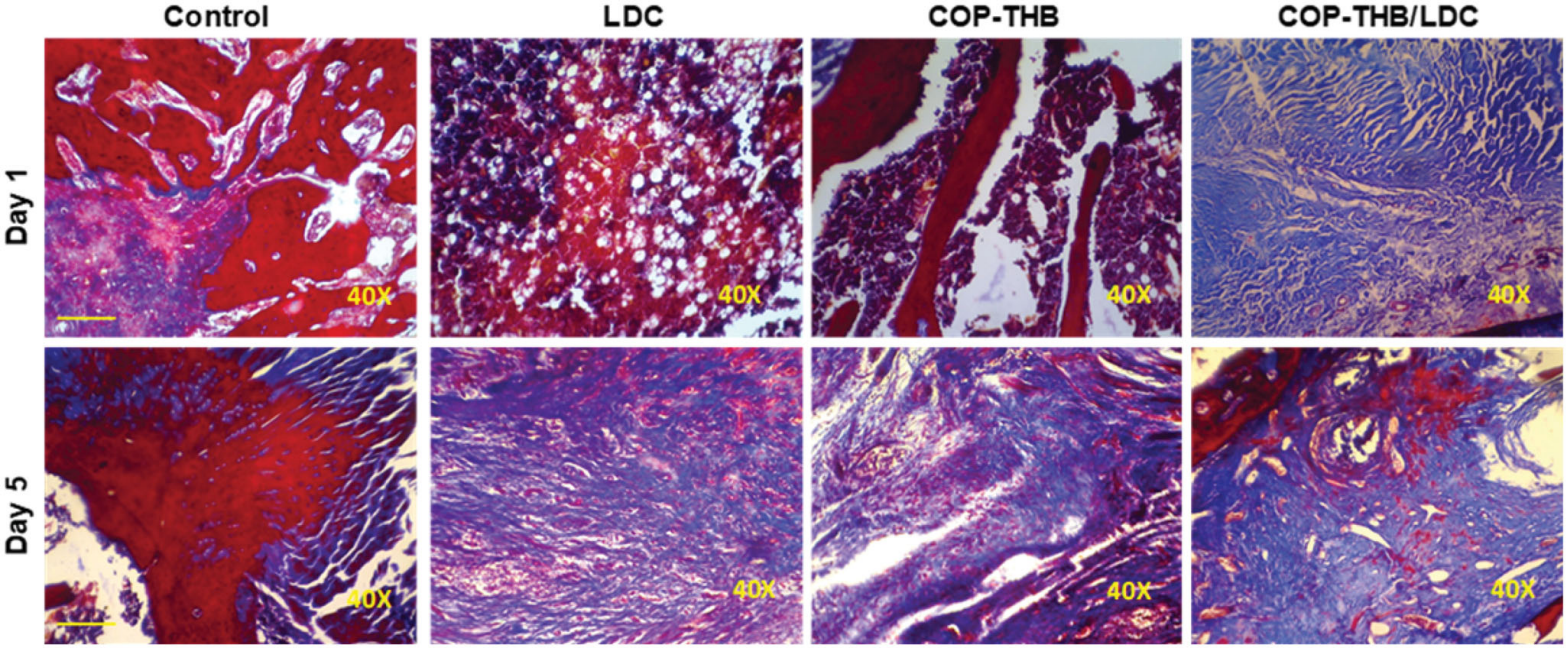
Preliminary safety evaluation of COP–THB/LDC formulations. Rats were subcutaneously injected with COP–THB/LDC, COP–THB, or saline, then skin sections were prepared on days 1 and 5 after injection and stained with Masson's trichrome stain (MTS). Scale bar, 100 μm.

We performed a histological assessment for the tissues of the RHP showed that compared to the control group, COP–THB/LDC effectively decreased CGN-induced inflammation (Figures 10 and 11) (saline). Since the key side effect of drug formulations is discomfort at the local injection site, local dermal irritation in rats caused by a single subcutaneous COP–THB/LDC or COP–THB injection has been investigated (Turner et al., 2011). We found mild inflammation at both 5 and 10 days for all groups after administration of the anesthetic. Both treated rats displayed normal feeding and behavior after injection and no pathology at the site of the injection (such as ulceration) or surrounding tissue was noted during the first 5 days after injection (Levoe et al., 2014; Bonnet et al., 2020). Inflammation in the control group after subcutaneous injection was not observed (Figure 12). As a result, the drug encapsulation lengthened the time that successful concentrations of the compounds remained at free nerve endings. However, even though hydrogels caused an increase in paw volume when compared to the control or hydrogel groups, this effect was less pronounced when compared to the carrageenan group, suggesting that hydrogel formulations did not cause potential local inflammatory effects, which is consistent with a previous study that showed histological evaluation after intramuscular injection of hydrogels (Cai et al., 2017; Guan et al., 2017). An acute inflammatory response to the soft tissue material of the drug carriers, injury from the medical operation or injection, or possibly acute local toxicity of LDC may all explain the observed acute inflammation on day 5. Since no inflammatory signs (redness and local heating) were reported after carrageenan intraplantar injection, the distinct increase in paw volume reported here can be due to the in-situ gelling properties of all formulations. To completely illustrate the mechanism of action, more rigorous safety testing, including systemic and local pharmacokinetic evaluation, will be needed. This indicates that the latest topical anesthetic formulations would be useful in the clinic.

### 3.9. *In vivo* tumor efficacy

**Figure 13.**
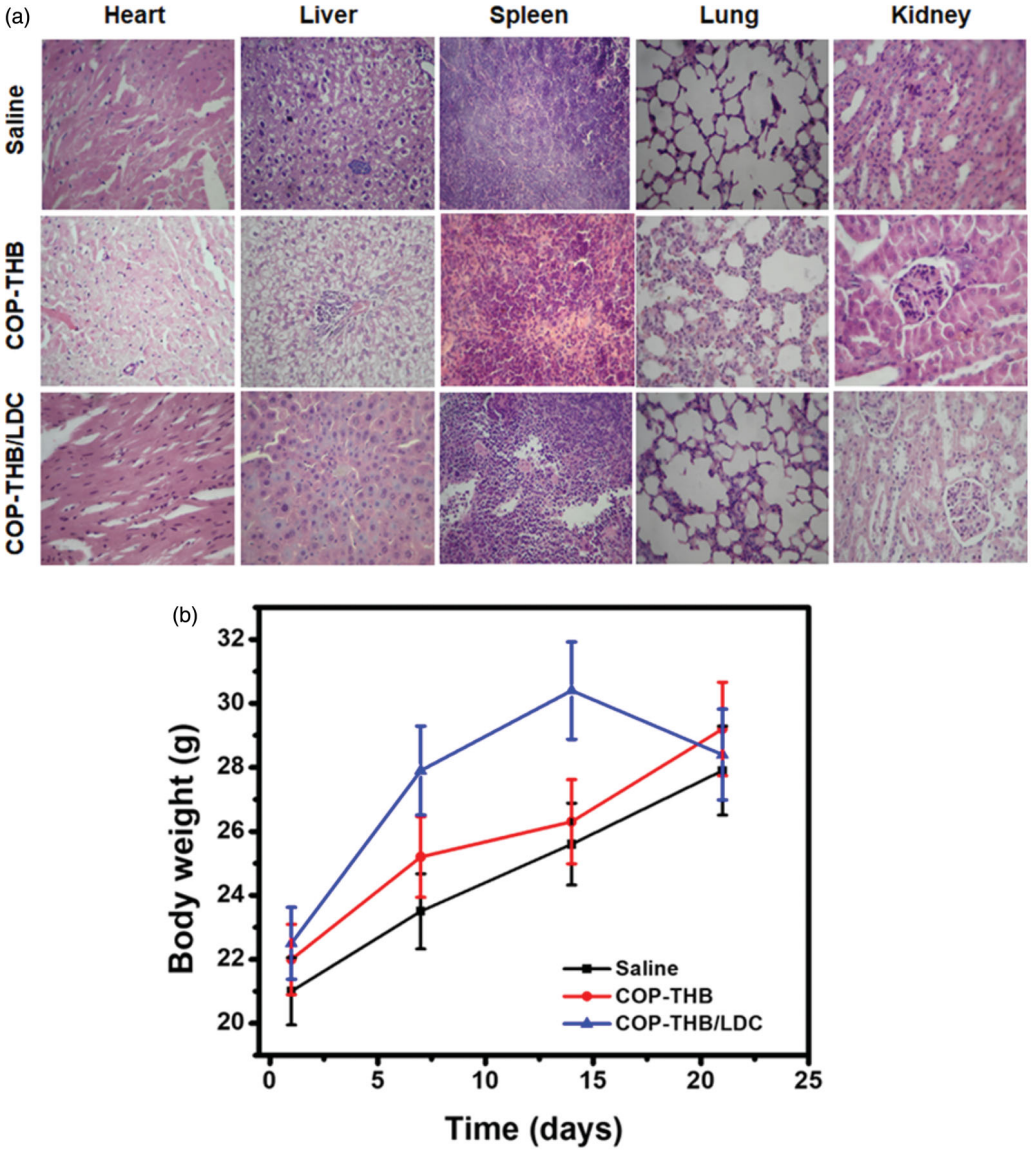
*In vivo* imaging and antitumor effect of COP–THB/LDC. H&E staining of major organs obtained from nude mice treated with saline and COP–THB/LDC for 3 weeks, respectively. There were no significant pathological lesions or damages in the major organs from mice that were treated with COP–THB/LDC. Scale bar: 100 μm (a). Bodyweight of mice after treatment with saline and COP–THB/LDC showed a gradually increased tendency, implying that COP–THB/LDC had good biocompatibility (*n* = 3) (b).

The most important aim of drug development is to create drugs with high anticancer efficacy and low toxicity (Zhang et al., 2020). We tested the drug carrier’s *in vivo* toxicity in mice treated with LDC-loaded COP–THB hydrogel via tail vein injection for safety reasons. The histological analysis of the spleen, kidney, and liver was performed using before LDC-loaded COP–THB hydrogel injection (control group) and after LDC-loaded COP–THB hydrogel injection (experimental group) as shown in Figure 13(a) (Shukla et al., 2021). Furthermore, no deaths were observed during the 48-h analysis. In both the control and LDC-loaded COP–THB hydrogel groups, no necrotic tubule abnormalities were found in kidney tissue. The liver and kidney structures of mice treated with the control and LDC-loaded COP–THB hydrogel groups were both standards. Spleen tissue structure was common in both the control and LDC-loaded COP–THB hydrogel classes, with no inflammatory changes. Furthermore, the RMSNs and saline groups both gained weight over 21 days in a similar pattern (Figure 13(b)) (You et al., 2017; Xie et al., 2018). Above all, the biocompatibility of the LDC-loaded COP–THB hydrogel can be summarized.

## 4. Conclusion

The polyvinylpyrrolidone with PVA cross‐linked with tetrahydroxyborate hydrogels by injection to achieve local anesthetics Lidocaine. The initial concentrations of COP and THB determine the mechanical and rheological properties of COP–THB hydrogels. The equilibrium cross-link density and physical properties of the resulting formulation are determined as a result of this. The COP–THB/LDC composite hydrogels were characterized by SEM and AFM with nanoscale range. When compared to free LDC, the release of LDC from the COP–THB hydrogels was slower and more prolonged, favoring both the anesthetic's length of action and toxicity reduction. These are the most appealing features of a local anesthetic device. The COP–THB/LDC hydrogels were substantially less toxic than the free drug in cytotoxicity studies, illustrating the defensive effect of encapsulation. When COP–THB/LDC hydrogels were locally delivered to mice with carrageenan-induced inflammatory in the inflamed RHP, the anesthetic effect was prolonged for several days. It's worth noting that COP–THB hydrogels with no ROS-scavenging activity had no anesthetic effect, which may be due to local damage caused by the injection site matrix. Furthermore, in a mouse model, COP–THB/LDC hydrogels completely suppressed *in vivo* tumor growth and effectively decreased *in vivo* toxicity. Hydrogels of COP–THB/LDC have a stronger anesthetic effect and lower toxicity than hydrogels of COP–THB and free LDC, according to *in vitro* and *in vivo* tests. These findings support the clinical use of this novel drug delivery system for safely extending and improving the local anesthetic effects.

## Supplementary Material

Supplemental Material
