# Supplementary material for: Facile design of lidocaine-loaded polymeric hydrogel to persuade effects of local anesthesia drug delivery system: complete in vitro and in vivo toxicity analyses
Source: Drug Deliv. 2021 Jun 11;28(1):1080–92. doi: 10.1080/10717544.2021.1931558 (PMC8204985; doi:10.1080/10717544.2021.1931558)
Supplement: Supplemental Material [file IDRD_A_1931558_SM6384.docx]

**Facile Design of Lidocaine loaded Polymeric Hydrogel to persuade effects of Local Anesthesia Drug Delivery system: Complete *In vitro* and *In vivo* Toxicity Analyses**

**Yan Li, Erxian Zhao, Li Li, Liying Bai, Wei Zhang***

Department of anesthesiology, The First Affiliated Hospital of Zhengzhou University, Zhengzhou 45000, Henan province, China.

***Corresponding author**

Dr. Wei Zhang

Department of anesthesiology, The First Affiliated Hospital of Zhengzhou University. NO.1, Jianshe Road, Zhengzhou 45000, Henan province, China.

**Mechanical properties**

The mechanical properties of the COP-THB hydrogels at room temperature were tested using a tensile tester (INSTRON 3365, Norwood, MA, USA). For tensile tests, hydrogels were cut into rectangular strips (25 mm × 15 mm × 3 mm, length × width × thickness), and the gauge length was 10 mm and the crosshead speed set at 10 mm/min. Compression stress was defined as the stress at a strain of 80%. Tensile and compressive experiments were performed at least 3 times for each sample. To test the tensile properties, the hydrogels were formed into a dumbbell shape with a length of 20 mm, a thickness of 1.5 mm and a width of 4 mm.

**Rheological analysis**

The specimens were tested with a TA Instruments ARES-LS2 rheometer (TA Instruments, New Castle, DE) equipped with a 50 mm stainless steel upper plate and a 600 grit sandpaper peltier bottom plate (47185A51, McMaster-Carr, Elmhurst, IL) to prevent slippage in order to test the rheological behaviors of COP-THB hydrogels. The sample was deformed under various shear strains after the strain scanning test. In the frequency range of 0.01-100 Hz at the 8x10^-4^ strain, the storage modulus (G') and loss modulus (G") were reported. Within the linear viscoelastic area of the systems, this strain value was selected, previously calculated by strain sweep (0.1%-500%) tests carried out in the same experimental setting at the frequency of 1 Hz. All tests were carried out at room temperature.

**Water content determination**

The COP-THB hydrogels were placed in an oven at a constant temperature of 50°C and dried until the weight of the COP-THB hydrogels remained constant. The water content W was calculated by the following equation (2) :

W = (m- m_0_)/m ……. (2)

where, m and m_0_ represent the mass of the COP-THB hydrogels before and after drying respectively. All samples were determined three times.

**Swelling ratio**

At room temperature, the swelling of COP-THB hydrogels in water has been studied. The hydrogels (W_0_) were weighed in a lyophilized state (before swelling) and after various swelling periods. At fixed time intervals, samples were weighed (W_t_) and the excess water was collected by putting the samples on the filter paper. Using the equation (1) to determine the swelling ratio of COP-THB hydrogels.

Swelling ratio (%) = [(W_t_ – W_0_)/W_0_] x 100% …….. (1)

where W_t_ is the weight of the swelling COP-THB hydrogels at different times, W_0_ is the initial weight. The swelling kinetics was analyzed from the experimental data of W(t)–t

**AFM analysis of COP–THB/LDC**

A MFP-3D-Bio (Asylum Testing, CA, USA) with cantilever 'HQ:NSC15/Al-BS' (μMesch) with 40 nN/nm stiffness in AC mode was used to measure the morphology of COP-THB and COP-THB hydrogel loaded with LDC (tapping mode). On mica surfaces, samples were deposited, and overnight dried. The speed of the scan was equal to the distance and the frequency of the scan was 0.6 Hz. The morphology of the COP-THB/LDC was examined at time zero and after 90 days of storage, and before and after the in vitro LDC release kinetics assays.

**SEM analysis of COP–THB/LDC**

The morphology of the COP-THB and COP-THB/LDC hydrogels was obtained by scanning electron microscopy (SEM) (S-4700, Hitachi Limited, Japan) using 15 kV for electron beam scanning using a sputter coater after coating with Au. On the cross-section of the hydrogels dried by lyophilization and cut with a sharp razor blade, the SEM measurements were made. Using an ImageJ application, SEM samples were analyzed.

**Drug load and release property**

Using phosphate buffer solution (pH 7.4) as a receptor, the in vitro release of LDC from COP-THB hydrogels was determined. The freshly prepared COP-THB hydrogels incorporating LDC were completely immersed in 50 mL of phosphate buffer solution for 30 minutes, then the soaking solution was aspirated and the LDC content (0.5, 1.0 and 2.0% w/w) in the soaking solution was calculated to determine the efficiency of drug encapsulation (DEE) and the efficiency of drug loading (DLE). With a circulating water bath, the 50 mL of PBS solution with soaked LDC-loaded COP-THB hydrogel was held at 37°C and stirred at 100 rpm with a magnetic stirring bar. Then, at intervals, 1.0 mL of the solution was taken and then 3.0 mL of brilliant blue Coomassie solution was applied. Subsequently, in the vial, 3.0 mL of fresh phosphate buffer solution was added and stirred until the next interval in the shaking incubator. A Shimadzu UV-1601 spectrophotometer at 280 nm analyzed the LDC release content of the solution and reported the data to measure the cumulative release rate (CRR) of the LDC-loaded COP-THB hydrogel system. To remove the effect of the volume on the sustained release of the drug, 1 mL of PBS solution was applied. The sum of three determinations reflects each data point. The calculation were as the followings equations (1,2, and 3):

DEE = m-m_1_/m × 100% …… (1)

DLE = m/M × 100% …….. (2)

$CRR=\frac{{50C}_{i}+\sum C(i-1)}{m} \times100\%$…….. (3)

where m is the total mass of LDC in the LDC-loaded COP-THB hydrogel, m_1_ is the mass of LDC in the LDC-loaded COP-THB hydrogel soaking solution, M is the mass after drying of the LDC-loaded COP-THB hydrogel, and C_i_ is the recorded concentrations of LDC at intervals.


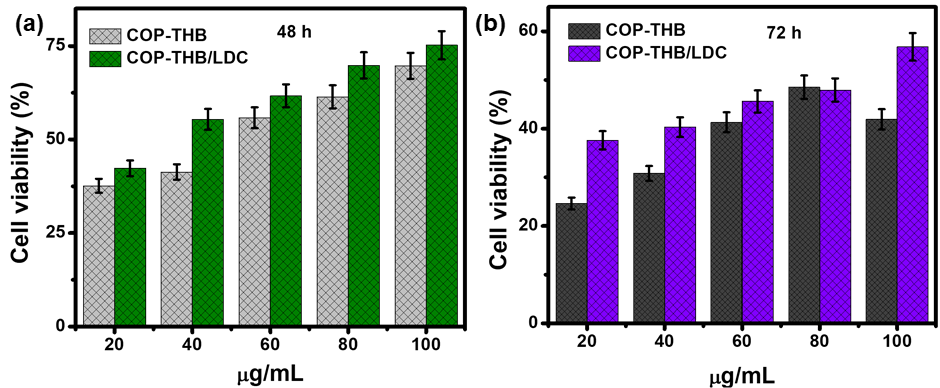


**Figure S1**. (a) Cell viability (MTT assay) of mouse fibroblast L929 cells after (a) 48 h and (b) 72 h exposure to various concentrations of COP-THB and COP-THB/LDC hydrogels ranging from 20 to 100 μg/mL. Data are presented as means ± standard deviation (n = 3).
